# Gene coexpression analysis reveals key pathways and hub genes related to late-acting self-incompatibility in *Camellia oleifera*

**DOI:** 10.3389/fpls.2022.1065872

**Published:** 2023-01-24

**Authors:** Chang Li, Yi Long, Mengqi Lu, Junqin Zhou, Sen Wang, Yan Xu, Xiaofeng Tan

**Affiliations:** ^1^ Key Laboratory of Cultivation and Protection for Non-Wood Forest Trees, Ministry of Education, Central South University of Forestry and Technology, Changsha, China; ^2^ Academy of Camellia Oil Tree, Central South University of Forestry and Technology, Changsha, China; ^3^ The Belt and Road International Union Research Center for Tropical Arid Non-wood Forest in Hunan Province, Changsha, China

**Keywords:** *camellia oleifera*, late-acting self-incompatibility, transcriptome, pollination, hub genes

## Abstract

**Introduction:**

Self-incompatibility (SI) is an important strategy for plants to maintain abundant variation to enhance their adaptability to the environment. *Camellia oleifera* is one of the most important woody oil plants and is widely cultivated in China. Late acting self-incompatibility (LSI) in *C. oleifera* results in a relatively poor fruit yield in the natural state, and understanding of the LSI mechanism remains limited.

**Methods:**

To better understand the molecular expression and gene coexpression network in the LSI reaction in *C. oleifera*, we conducted self- and cross-pollination experiments at two different flower bud developmental stages (3–4 d before flowering and 1 d before flowering), and cytological observation, fruit setting rate (FSR) investigation and RNA-Seq analysis were performed to investigate the mechanism of the male −female interaction and identify hub genes responsible for the LSI in *C. oleifera*.

**Results:**

Based on the 21 ovary transcriptomes, a total of 7669 DEGs were identified after filtering out low-expression genes. Weighted gene coexpression network analysis (WGCNA) divided the DEGs into 15 modules. Genes in the blue module (1163 genes) were positively correlated with FSR, and genes in the pink module (339 genes) were negatively correlated with FSR. KEGG analysis indicated that flavonoid biosynthesis, plant MAPK signaling pathways, ubiquitin-mediated proteolysis, and plant-pathogen interaction were the crucial pathways for the LSI reaction. Fifty four transcription factors (TFs) were obtained in the two key modules, and WRKY and MYB were potentially involved in the LSI reaction in *C. oleifera*. Network establishment indicated that genes encoding G-type lectin S-receptor-like serine (lecRLK), isoflavone 3’-hydroxylase-like (CYP81Q32), cytochrome P450 87A3-like (CYP87A3), and probable calcium-binding protein (CML41) were the hub genes that positively responded to the LSI reaction. The other DEGs inside the two modules, including protein RALF-like 10 (RALF), F-box and pectin acetylesterase (MTERF5), might also play vital roles in the LSI reaction in *C. oleifera*.

**Discussion:**

Overall, our study provides a meaningful resource for gene network studies of the LSI reaction process and subsequent analyses of pollen−pistil interactions and TF roles in the LSI reaction, and it also provides new insights for exploring the mechanisms of the LSI response.

## 1 Introduction

The Camellia oil tree (*Camellia oleifera* Abel.), one of the four major woody oil tree species in the world, is widely cultivated as an oil crop in many countries, such as China, India, Brazil, Japan, the Philippines and South Korea (Chen et al., 2017; Lin et al., 2022b). Camellia oil is a high-grade edible oil that scavenges free radicals and prevents cardiovascular disease and has been used as an edible oil for more than 2300 years in China (Quan et al., 2022). The unsaturated fatty acid content in Camellia oil reaches 90%, and Camellia oil also contains sterols, squalene, polyphenols, sterols, vitamin E and other compounds that are good for human health (Lee and Yen, 2006; Hosokawa et al., 2013; Zhang et al., 2021). It is widely used in products such as ink, lubricants, and cosmetics (Lin et al., 2018). Although *C. oleifera* has been cultivated on approximately 3 million hectares, 70% of its planting area is low-yield, and the annual production cannot meet the demand for this healthy oil (Hajjar and Hodgkin, 2007). The main reason for the low yield of *C. oleifera* is its self-incompatibility (SI) property and the improper configuration of pollination trees. Thus, it is meaningful to study the SI mechanism of *C. oleifera* to improve its seed yield.

SI is a complex reproductive barrier mechanism between organisms that is critical to speciation (Broz and Bedinger, 2021), and it has been widely adopted by flowering plants to promote cross-pollination and prevent self-pollination (Wu et al., 2020b). The SI mechanism has been extensively studied and can be divided into three types: sporophytic self-incompatibility (SSI), gametophytic SI (GSI) and late-acting self-incompatibility (LSI) (Zhou et al., 2020). In SSI, such as in Asteraceae and Convolvulaceae, the SI reaction was determined by two polymorphic S loci that were genetically linked: pollen-coat-specific cysteine-rich protein (SCR) and stigma-specific serine/threonine receptor kinase (SRK), which could inhibit the germination and growth of self-pollen (Wang et al., 2014; Nasrallah, 2019). In GSI, such as in Solanaceae and Rosaceae, S-specificity was determined by haploid pollen (Duan et al., 2020) and controlled by a single S-locus composed of two linked polymorphic genes: pollen-specific F-box protein (SFB/SLF) and style-specific T2 ribonuclease (S-RNase) (Gordillo-Romero et al., 2020). Another GSI mechanism observed in *Papaver rhoeas* showed that incompatible pollen could trigger the interaction between the male S-loci *PrpS* and the female S-loci *PrsS*, which leads to Ca^2+^ signaling cascades and initiates programmed cell death (PCD) (Wheeler et al., 2009; Eaves et al., 2014; Bedinger et al., 2017). LSI has been found in many plants, such as *C. sinensis*, *Nymphaea* spp. and *Theobroma cacao*, in which the pollen tube can grow successfully in the styles, but the inhibition occurs either prezygotically or postzygotically (Hao et al., 2012). Many studies have been performed to identify the key factors involved in the LSI response, but the mechanism remains unclear (Chen et al., 2012; Sun et al., 2019). The genetic basis of LSI was hypothesized to be gametophytic under genetic control (Zhou and Zheng, 2015; Zhang et al., 2016). However, no effort has been made to identify the genes of SI loci.

The SI reaction is a relatively complex signal transduction process. In addition to the S factor, non-S factors are also required during the recognition between pollen and pistils (McClure et al., 2011). In *Petunia hybrida*, the S-RNase binding protein (SBP1) from pollen could bind to *S-RNase*, and it was proposed that *SBP1* can form a complex with SLF and allow nonself S-RNase degradation. SBP1 can also interact with other non-S factors, such as arabinogalactan proteins (AGPs) and 120-kDa glycoprotein (120K) (O’Brien et al., 2004; Ben-Naim et al, 2006). The participation of thioredoxin h proteins (THL) was involved in pollen rejection, where the downregulation of *THL1* could lead to the breakdown of SI (Haffani et al., 2004). In *Nicotiana*, suppressing *HT-B* expression in the pistil could prevent S-specific pollen rejection (Covey et al., 2010). Moreover, there were many other identified non-S factors, such as calcium-binding protein (*CML*) (Miao et al., 2013), actin-binding proteins (*ABPs*) (Poulter et al., 2010), *RALF* (Bedinger et al., 2010) and actin-depolymerizing factor (*ADF*) (Dong et al., 2021).

In *C. oleifera*, recent studies showed that pollen tubes in self-pollinated pistils could grow normally through the style but failed to complete double fertilization (He et al., 2020; Zhou et al., 2020). Several candidate genes and pathways have been found that might participate in the LSI reaction by comparing the transcriptomes of self- and cross-pollination (He et al., 2020). However, the molecular mechanism of the LSI in *C. oleifera* remains unclear. In this study, we investigated the fruit setting rates under self- and cross-pollination in two different developmental stages of flower buds, observed the differences in pollen tube growth between self- and cross-pollination, and performed transcriptome sequencing. Furthermore, weighted gene coexpression network analysis (WGCNA) was used to identify the hub genes and transcription factors (TFs) related to the LSI response of *C. oleifera*. This study provides new insights into the mechanisms of the LSI response and is valuable for improving the yield and haploid breeding of *C. oleifera.*

## 2 Materials and methods

### 2.1 Plant materials

The *C. oleifera* cultivars ‘Hua Jin’ (HJ) and ‘Hua Xin’ (HX) were used in this study. They were selected from among 84 C*. oleifera* clones by a comparative regional assessment and were identified by Professors Tan Xiaofeng and Yuan Deyi at Central South University of Forestry and Technology (Zhang et al., 2021). The cultivar was planted in Huju Forest Farm in the Chaling district, Zhuzhou city, Hunan Province, China (26^°C^47’24’ N, 113^°C^32’24’ E). During the peak flowering period of HJ in October, flower buds at different developmental stages were selected for pollination, including 3-4 d before flowering (stage 3), 1 d before flowering (stage 1) and 1 d after flowering (stage 0).

### 2.2 Pollination treatment and sample collection

We designed six treatments for pollination, including self-pollination at stage 3 (SPT3), self-pollination at stage 1 (SPT1), cross-pollination at stage 3 (CPT3), self-pollination at stage 1 (CPT1), natural pollination (NPT0), no pollination at stage 1 (CKT1), and no pollination at stage 3 (CKT3). Among them, self-pollination was based on HJ as the parent, cross-pollination was based on HJ as the female parent, and HX as the male parent. Healthy and strong plants at the same growth stage were selected as female parents for pollination, and 500 flowers in different ten trees were pollinated in each treatment. Before artificial pollination, mature pollen of HJ and HX was collected from anthers in advance during the bud stage, placed in sulfate paper bags and kept at 25°C for 8 h. The experiment was conducted at midmorning on sunny days. The flower buds at the correct developmental stage were selected, pollinated before removing the corolla and anthers, and then bagged in sulfate paper bags. At 48 h after pollination, seven groups of samples for RNA-seq were collected from the same ten trees, and each group contained three biological replicates. Each replicate contained 30 ovaries. The collected samples were immediately placed into liquid nitrogen for preservation. In addition, thirty pistils for pollen tube observations were collected at different intervals (0, 12, 24, 36, 48, 60, 72, 84 h) after pollination from the same ten trees and then stored in Carnoy’s fixative.

### 2.3 Cytological observation, stigma acceptability test and fruit set survey

The fixed pistils were macerated with sodium hypochlorite for 2 h to soften and were then washed with deionized water and macerated in 8 M NaOH for 2 h. Then, the pistils were stained with 0.1% aniline blue for 6 h. After staining, the styles were split along the vertical axis into 3-5 sections with a surgical blade. Finally, we tracked pollen tube growth using fluorescence microscopy (Nikon Instech Co., Ltd., Kawasaki, Japan).

Pollen viability was determined by benzidine staining. Fresh style sections were immersed in a reaction solution containing benzidine-hydrogen peroxide (1% benzidine: 3% hydrogen peroxide: water = 4:11:22, v/v) for observation. If the styles were receptive, the reaction solution and style gradually turned brown or black as the active pollen. The fruit setting rate was investigated 60 d after pollination.

### 2.4 Transcriptome sequencing and alternative splicing analysis

The total RNA of samples was extracted using TRIzol reagent (Invitrogen, Carlsbad, CA, USA) according to the manufacturer’s protocol, and rRNA was subsequently removed using a Ribo-Zero™ Magnetic Kit (Epicenter, Madison, WI, USA). Then, the cDNA fragments were purified with a QIAquick PCR extraction kit (Qiagen, Venlo, The Netherlands), end-repaired, polyadenylated, and ligated to Illumina sequencing adapters. The ligation products were subjected to size selection *via* agarose gel electrophoresis, amplified *via* PCR, and sequenced using the Illumina NovaSeq 6000 platform.

Reads obtained from the sequencing machines included raw reads, which affected the subsequent assembly and analysis. To obtain high-quality clean reads, the reads were further filtered by fastp (Chen et al., 2018), and rRNA sequences were removed by alignment. The genome data of *C. oleifera* ‘Huashuo’ have been obtained (unpublished data). An index of the reference genome was built, and the paired-end clean reads were mapped to the reference genome using HISAT 2.2.4 (Kim et al., 2015) with “-rna-strandness RF” and other parameters set as a default. The mapped reads of each sample were assembled by using StringTie v1.3.1 (Pertea et al., 2015) in a reference-based approach. Based on the comparison results of all the reads (Total_Mapped reads) that can be located on the genome, we calculate the distribution of reads in the reference genome. We divided the regions mapped to the genome into exon, intron and intergenic regions. The unannotated transcripts were blasted against databases including, including the NR, Swiss-Prot, GO and KEGG databases, the whole pineline of the read assembly, alignment and annonation were present as a flowchart (**Supplementary Figure S1A**). RNA differential expression analysis was performed by DESeq2 (Love et al., 2014) software between two different groups (and by edgeR (Robinson et al., 2010) between two samples), and the genes with the parameters of false discovery rate (FDR) ≤ 0.05 and absolute fold change ≥ 2 were considered differentially expressed genes.

The software rMATS (Shen et al., 2014) (version 4.0.1) (http://rnaseq-mats.sourceforge.net/index.html ) was used to identify alternative splicing events and analyze differential alternative splicing events between samples. We identified AS events with a false discovery rate (FDR) <0.05 in a comparison as significant AS events.

### 2.5 Weighted gene coexpression network analysis

To identify the hub genes related to the LSI reaction in *C. oleifera*, weight gene coexpression network analysis (WGCNA) was performed on the OmicShare platform (https://www.omicsmart.com/WGCNA/home.html#/taskPandect) based on the differentially expressed genes (DEGs) and fruit setting rates. The DEGs with FPKM values < 1 in all samples were filtered out before WGCNA analysis (Lin et al., 2022a), so as to improve the accuracy of network construction. A total of 7669 DEGs were obtained to construct a Gene hierarchical cluster diagram based on the correlation of gene expression, coexpression modules were constructed with a soft threshold of 8.0, mini-num module size of 50, and Similarity of module eigenvalue of 0.9 for merging similar transcripts. The key modules were selected based on the correlation coefficients between traits and modules, and the gene coexpression network was constructed based on the top 10% of DEGs ordered by the edge weight coefficient within the module. Pearson correlation coefficients between the hub genes and TFs of each module, include blue module and pink module, were computed by OmicShare tools (https://www.omicshare.com/tools). Regulatory networks were visualized by Cytoscape 3.7.2 (Shannon et al., 2003). The whole process of the WGCNA was present as a flowchart (**Supplementary Figure S1B**).

### 2.6 Quantitative real-time reverse transcription PCR analysis

To verify the accuracy of the RNA-seq data, a total of 12 DEGs related to SI were selected and evaluated by qRT−PCR. The total RNA of the 21 samples in seven comparison groups (NPT0, CKT3, CKT1, SPT1, SPT3, CPT1, CPT3) was extracted by the Goldenstar™ RT6 cDNA Synthesis Kit Ver.2 (Beijing TsingKe Biotech Co., Ltd, China), and the remaining elimination of potential gDNA and reverse transcription were performed with HiScript^®^ II Q RT SuperMix for qPCR (+gDNA wiper) (Vazyme, Nanjing, China). Specific primers for the 12 DEGs were designed by Primer Premier 5.0 (**Supplementary Table S1**). The qRT−PCR was conducted in conjunction with ChamQ Universal SYBR qPCR Master Mix #Q711 (Vazyme, Nanjing, China). The experiment was performed on the CFX96 Real Time PCR System (Bio-Rad, Hercules, CA, USA), and *CoGAPDH* (KC337052.1) was chosen as the reference gene for Camellia oil tree. All analyses were repeated with three biological replicates, and the relative expression level of 12 DEGs were calculated with the 2^-ΔΔCt^ method. The results were analyzed using Microsoft Office Excel 2013 (Microsoft, Redmond, WA, USA) and SPSS (ver. 20.0; SPSS, Inc., Chicago, IL, United States) software.

## 3 Results

### 3.1 The stigma of immature flower buds (3 DBFs) exhibiting high acceptability in *C.oleifera*

**Figure 1:**
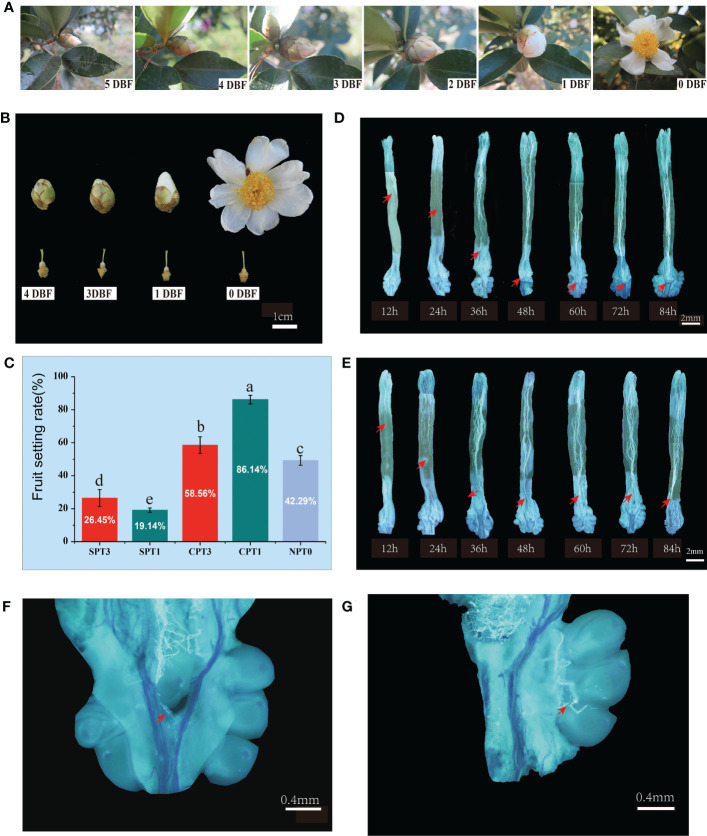
Investigation of fruit setting rates and observation of pollen tube growth in pistils in *Camellia oleifera* HJ. **(A)** Observation of flower bud development in nature; 5 DBF: 5 days before flowering, 4 DBF: 4 days before flowering, 3 DBF: 3 days before flowering, 2 DBF: 2 days before flowering, 1 DBF: 1 day before flowering. **(B)** Flower buds at different developmental stages. **(C)** Fruit setting rates of SPT1, SPT3, CPT1, CPT3 and NPT0. **(D)** Pollen tube growth from 12 h to 84 h in the pistil after cross-pollination. **(E)** Pollen tube growth from 12 h to 84 h in the pistil after self-pollination. **(F, G)** Successful pollen tube growth to the ovary 60 h after self-pollination.

The external morphological characteristics of flower buds of *C. oleifera* were observed and recorded starting at 6 d before flowering (DBF) to determine the exact flower bud stage for bud pollination (**Figures 1A, B**). The *C. oleifera* flower buds began to swell and entered the middle bud stage three days before flowering. Self-pollination at the early bud stage could reduce the reaction of self-incompatibility, promote the fruit setting rate and help to create inbred offspring in many plants with SI properties (Zhang et al., 2019). Since then, immature flower buds (3 DBFs) and mature flower buds (1 DBF) were both selected for self- and cross- pollination in this study.

The acceptability of stigmas greatly affects the efficiency of hybrid pollination. To test the stigma receptivity of the four main flower development stages of *C. oleifera*, the most reliable method, called the benzidine staining method, was used in this study. The results showed that all stigmas from the four flower development stages were receptive, as the stigmas were bubbly and brown, and the style was the most receptive on the day of flowering. The color of the stigma became dark brown, and there were more bubbles (**Supplementary Figure S2A**).

### 3.2 Transcriptome profiles of 21 RNA libraries from camellia oil tree ovaries pollinated with different treatments

Self- and cross-pollination were performed on pistils at 3 DBF and 1 DBF. The fruiting rates were investigated 60 d after pollination. The results showed that there was a significant difference in the fruit setting rate between self-pollination and cross-pollination, regardless of pollination at earlier or later developmental stages. The fruiting rates of CPT1 and CPT3 reached 86.14% and 58.56%, respectively, which were significantly higher than those of SPT1 (19.14%) and SPT3 (26.45%). Moreover, the early natural fruit setting rates of *C. oleifera* were relatively moderate, with a fruit setting rate of 49.29% (**Figure 1C**). The results indicated that the pollen from different varieties was more easily accepted by pistils than by self-pollen. Furthermore, the fruit setting rate of SPT3 was higher than that of SPT1, and the fruit setting rate of CPT1 was significantly higher than that of CPT3. This suggested that the transcription level and translation level of SI-related genes in 3 DBF buds were lower than those in 1 DBF bud, and mature pistils were more beneficial to the successful germination, growth and effective fertilization of cross-pollinated pollen tubes.

To evaluate the pollen germination rates and pollen tube growth between self- and cross-pollination and to determine the actual time when the self-incompatibility reaction occurred after self-pollination, fluorescence microscopic observation of pollen tube growth was performed (**Figures 1D, E**). The results showed that there were no differences in pollen tube growth in pistils at different developmental stages. The pollen grains germinated normally at 12 h after self- and cross-pollination. At 48 h after pollination, all of the pollen tubes grew smoothly to the upper part of the ovary. Thereafter, the self-pollinated pollen tubes stopped growing with the features of tissue distortion and folding, which are characteristic of programmed cell death (PCD). The cross-pollinated pollen tubes continued to grow downward until the double fertilization reaction was completed at approximately 72 h after cross-pollination (**Figures 1F, G**). The cytological observation showed that the SI of Camellia oil tree belonged to the LSI. Moreover, these results supported the SI reaction occurred at 48h of self-pollination, which was the best sampling duration for transcriptome sequencing.

### 3.3 Transcriptome sequencing revealed differentially expressed genes related to LSI in *C. oleifera*

**Figure 2:**
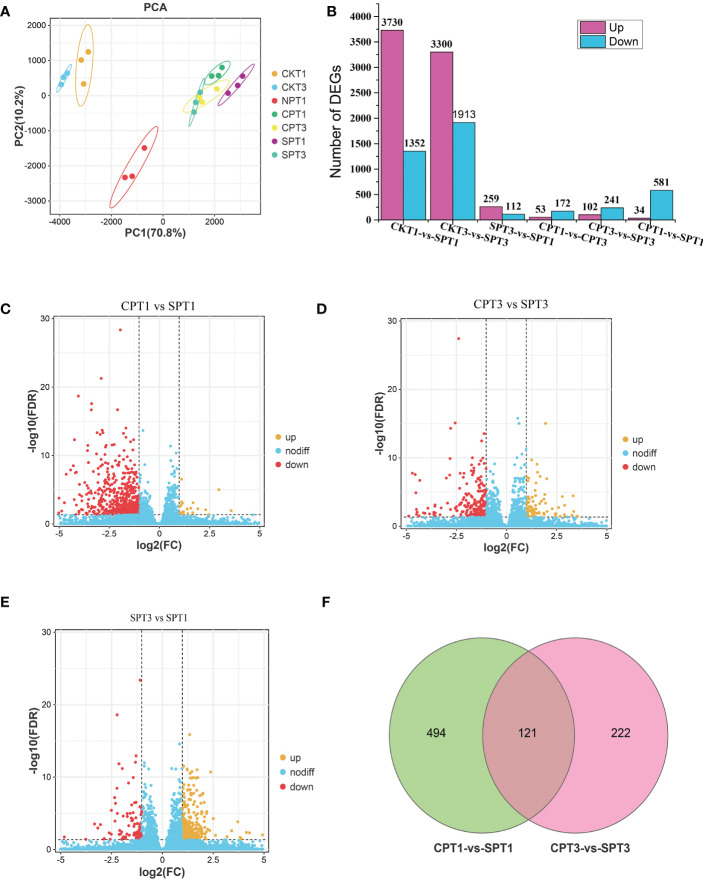
Graphical representation of DEGs related to late-acting self-incompatibility in different treatments of *Camellia oleifera* ‘Huajin’. **(A)** PCA score plot. **(B)** Number of up- and downregulated DEGs among the six gene sets. **(C)** Volcano plot of all DEGs in CPT1 vs. SPT1. **(D)** Volcano plot of all DEGs in CPT3 vs. SPT3. **(E)** Volcano plot of all DEGs in SPT3 vs. SPT1. **(F)** The Venn diagram represents the number of overlapping DEGs between self- and cross-pollination.

To further explore the molecular mechanism of LSI of *C. oleifera*, comparative transcriptomic sequencing analyses of the ovaries after different pollination treatments (SPT3, SPT1, CPT3, CPT1, NPT0, CKT1, CKT3) were performed. A total of 1.15 GB of high-quality data were obtained from the 21 cDNA libraries, with an average of 54.97 MB of data per sample. The Q30 percentages were greater than 93.19%, while the GC percentage of each sample was approximately 43% (**Table 1**). Approximately 87.82–91.03% of reads were mapped to the genome of *C. oleifera*. A total of 125,475 genes were identified including 1,5091 novel genes. The DEGs related to the LSI of Camellia oil tree were identified by the FPKM values from each sample library, and the expression levels of genes in the ovaries after compatible and self-incompatible pollination were assessed. The high reproducibility of the replicates was shown in a PCA plot (**Figure 2A**). Among these samples, there was a significant difference in FSR under each pollination treatment, and the FSR under cross-pollination was significantly higher than that under self-pollination. Next, 21 samples were divided into six groups for analysis (CKT1 vs. SPT1, CKT3 vs. SPT3, SPT3 vs. SPT1, CPT1 vs. CPT3, CPT3 vs. SPT3, CPT1 vs. SPT1), and the number of DEGs for the six groups is shown in **Figure 2B**. Among these comparisons, the CKT3 vs. SPT3 group had the most DEGs, reaching 5213 (3300 upregulated, 1913 downregulated). Next, there were 5082 DEGs (3730 upregulated, 1352 downregulated) in the CKT1 vs. SPT1 group. However, only 371, 225, 343, and 615 DEGs were identified in the SPT3 vs. SPT1, CPT1 vs. CPT3, CPT3 vs. SPT3, and CPT1 vs. SPT1 comparisons, respectively (**Figures 2C–E**). The comparison analysis showed that 121 DEGs overlapped among CPT1 vs. SPT1 and CPT3 vs. SPT3 (**Figure 2F**). The results suggested that these DEGs in six comparison groups should be involved in LSI reaction of *C. oleifera*.

**Table 1:** Quality of the camellia oil tree ovary transcriptome.

Sample	Sample codes	Total raw reads	Total clean reads	Q30 (%)	GC (%)	Unique_Mapped (%)	Mapped reads (%)
NPT0	A1	62,482,212	62,247,734	94.21%	43.16%	36396173 (58.51%)	67229784 (89.60%)
	A2	67,112,546	66,854,026	93.81%	42.98%	38944077 (58.29%)	59878759 (89.62%)
	A3	64,084,860	63,811,526	93.40%	43.16%	37482949 (58.79%)	62141099 (90.09%)
CPT1	B1	50,592,626	50,494,404	94.48%	43.53%	29922315 (59.30%)	55999174 (90.02%)
	B2	44,830,768	44,751,078	94.48%	43.20%	26668556 (59.64%)	57581027 (90.31%)
	B3	53,054,368	52,959,248	94.56%	43.46%	31402039 (59.33%)	55522352 (90.04%)
CPT3	C1	44,731,962	44,651,664	94.45%	43.27%	26364601 (59.09%)	60356399 (90.81%)
	C2	51,850,544	51,762,268	94.90%	43.32%	29931995 (57.87%)	46041841 (89.01%)
	C3	44,051,500	43,967,932	94.39%	43.40%	25252281 (57.47%)	38590971 (87.82%)
SPT1	D1	47,975,684	47,893,040	94.74%	43.45%	28096446 (58.71%)	47256978 (89.90%)
	D2	50,427,968	50,328,600	94.26%	43.55%	29748589 (59.14%)	49956565 (90.47%)
	D3	52,694,372	52,598,992	94.04%	43.50%	30664191 (58.34%)	47859831 (90.28%)
SPT3	E1	43,587,054	43,510,186	94.34%	43.48%	25219730 (57.99%)	38480118 (88.48%)
	E2	51,270,600	51,179,956	94.59%	43.47%	30024891 (58.70%)	48050770 (90.79%)
	E3	47,628,512	47,541,512	94.65%	43.43%	27944148 (58.82%)	46323594 (90.57%)
CKT1	F1	66,696,438	66,499,570	94.16%	43.09%	39267036 (59.08%)	45538893 (90.53%)
	F2	69,220,840	69,024,474	94.41%	43.21%	40222163 (58.31%)	45928274 (91.03%)
	F3	61,923,614	61,709,808	93.82%	42.94%	36100019 (58.54%)	43346846 (90.57%)
CKT3	G1	75,329,912	75,083,268	94.05%	43.06%	43464853 (57.93%)	40116367 (89.91%)
	G2	53,221,902	53,050,914	93.97%	42.93%	31149033 (58.76%)	43222116 (90.98%)
	G3	55,413,742	55,258,746	94.15%	43.02%	32439111 (58.75%)	40560593 (90.71%)

### 3.4 GO and KEGG enrichment analysis of DEGs

**Figure 3:**
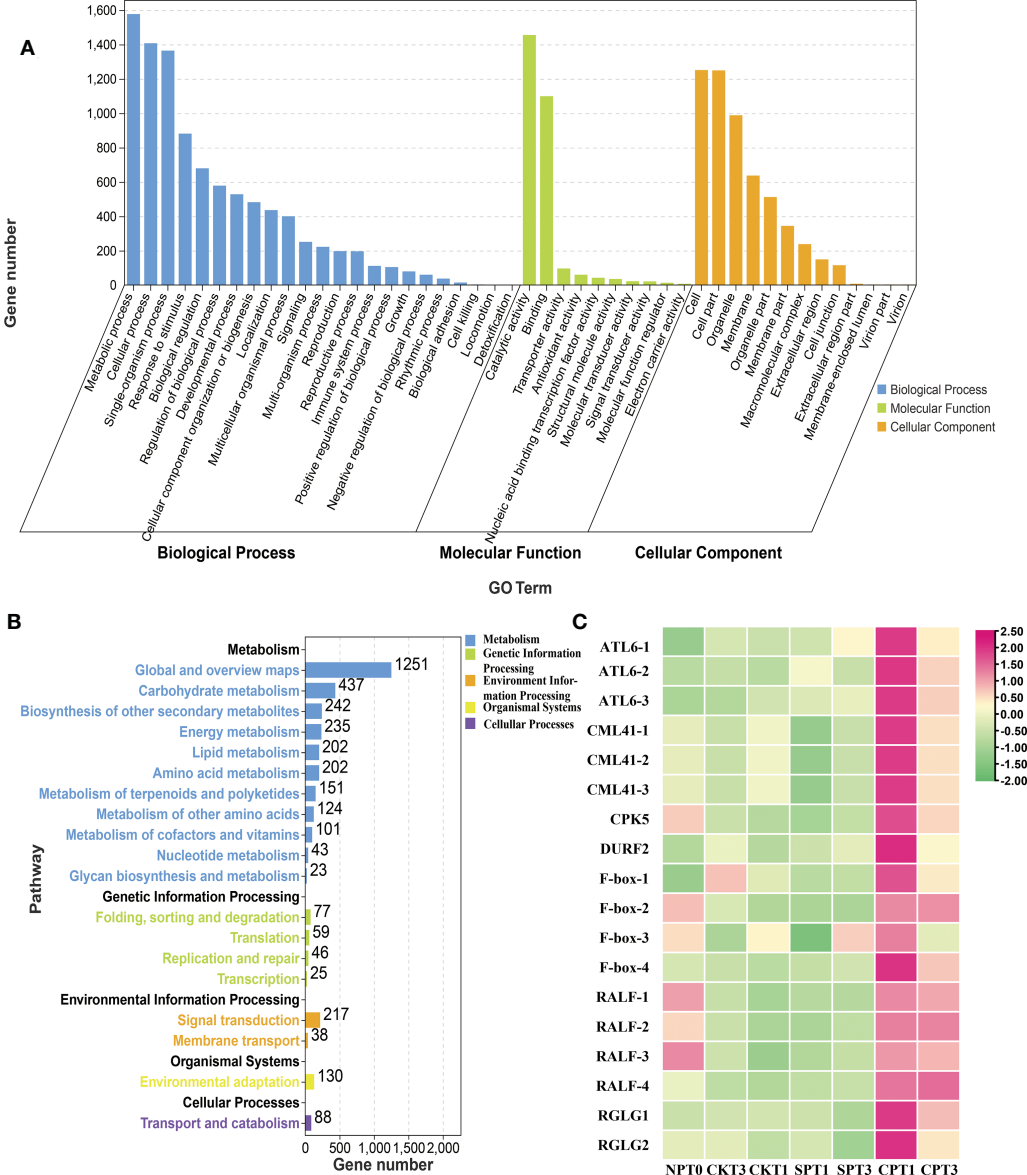
Analysis of DEGs in *Camellia oleifera* HJ. **(A)** GO enrichment analysis of the DEGs in *C. oleifera* HJ; **(B)** KEGG enrichment analysis of the DEGs in (C) oleifera HJ; **(C)** Expression profiles of crucial DEGs related to LSI in (C) *oleifera* HJ.

Gene Ontology (GO) enrichment analyses can help to determine which biological functions the DEGs are significantly associated with. For GO functional analysis, all the DEGs in the six comparison groups were divided into 53 functional groups in the molecular function (MF, 12 subcategories), cellular component (CC, 17 subcategories), and biological process (BP, 24 subcategories) categories. In the CC category, the terms that were significantly enriched were “cell”, “cell part”, “organelle” and “membrane”. In the MF category, the DEGs were enriched in “catalytic activity”, “binding” and “transporter activity”. In the BP category, “metabolic process”, “cellular process”, “single-organism process” and “response to stimulus” were more abundant than other terms (**Figure 3A** and **Supplementary Table S2**). For the DEGs in the six comparison groups, most GO terms were enriched in the BP and MF categories. Kyoto Encyclopedia of Genes and Genomes (KEGG) pathway analysis was conducted to gain insight into the actual biological function of DEGs. All the DEGs in the six comparison groups were significantly mapped to 37 KEGG pathways in five categories: metabolism, environmental information, genetic information processing, organismal systems, and cellular processing. The results showed that most DEGs identified were enriched in “Plant hormone signal transduction”, “Flavonoid biosynthesis”, “Brassinosteroid biosynthesis”, “ABC transporters”, “Ubiquitin mediated proteolysis” and “Stilbenoid, diarylheptanoid and gingerol biosynthesis”. Of note, the DEGs were significantly enriched in “plant hormone signal transduction” in all group comparisons except for CPT1 vs. CPT3 (**Figure 3B**; **Supplementary Figure S2B** and **Table S3**), especially in ethylene, brassinosteroid and jasmonic acid signal transduction pathways, indicating that DEGs enriched in plant hormone signal transduction was involved in the LSI reaction of Camellia oil tree. In addition, DEGs were significantly enriched in brassinosteroid biosynthesis in three groups (CPT1 vs. SPT1, CPT3 vs. SPT3 and CKT1 vs. SPT1), and DEGs were also significantly enriched in flavonoid biosynthesis in the other three comparison groups (CKT1 vs. SPT1, CKT3 vs. SPT3 and CPT1 vs. CPT3). Therefore, the results indicated that molecular and cellular events, including plant hormone signal transduction, brassinosteroid biosynthesis, flavonoid biosynthesis, etc., occurred in the LSI reaction in *Camellia* oil tree.

### 3.5 Co-expression network analysis identified key modules correlated with LSI reaction in *C. oleifera*

**Figure 4:**
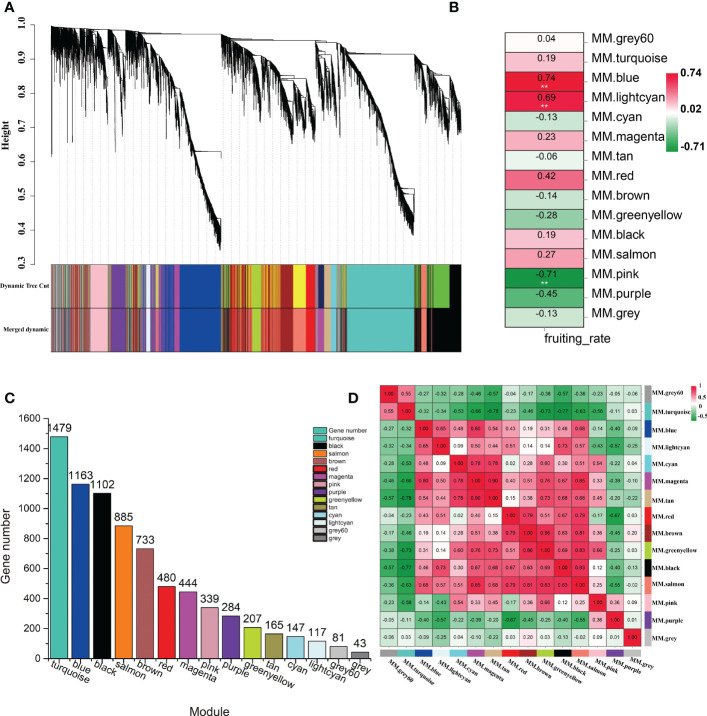
Identification of coexpression gene modules using differentially expressed genes. **(A)** Dendrogram of all filtered genes enriched according to a dissimilarity measure (1-TOM) and the cluster module colors. **(B)** Heatmap of the correlation between the fruit setting rates and module eigengenes (MEs) of bladder cancer. The redder the module color, the more significant the relationship. **(C)** Number of members in the 15 modules. **(D)** Module-sample correlation heatmap to intuitively visualize the expression pattern of each module in each sample.

To explore the specific genes and the genetic regulatory network in the LSI reaction in *C. oleifera* ovaries, WGCNA was performed based on 7669 DEGs, which with FPKM values < 1 in all samples were filtered out, among the six comparison groups and the fruit setting rate phenotypes (**Supplementary Table S4**). A cluster dendrogram was generated to cluster the genes with similar expression patterns into the same module (**Figure 4A**). A total of 15 coexpressed modules (M1–M15) were obtained after dynamic tree cutting and merging, and each module was marked with different colors, including module 1 (turquoise, 1,479 genes), module 2 (blue, 1,163 genes), module 3 (black, 1,102 genes), module 4 (salmon, 885 genes), module 5 (brown, 733 genes), module 6 (red, 480 genes), module 7 (magenta, 444 genes), module 8 (pink, 339 genes), module 9 (purple, 284 genes), module 10 (greenyellow, 207 genes), module 11 (tan, 165 genes), module 12 (cyan, 147 genes), module 13 (lightcyan, 117 genes), and module 14 (gray60, 81 genes) (**Figure 4C**). Forty-three genes did not belong to any of the modules and were assigned to the gray module. Moreover, the correlation between the fruit setting rate and the modules was analyzed, and the correlation coefficients between them ranged from -0.71 to 0.74. Among the 15 modules, three modules (M2, M13, and M8) had high correlation coefficients and showed significant associations (p < 0.05) with the fruit setting rates (**Figure 4B**). We also constructed a module-sample correlation heatmap to intuitively visualize the expression pattern of each module in each sample (**Figure 4D**).

### 3.6 Functional specific enrichment analysis of the two key modules

To further understand the specific functions of the key module, GO and KEGG enrichment analyses of the DEGs from the two key modules (blue and pink) were performed. GO analysis revealed that “response to stress”, “response to endogenous stimulus”, “hormone-mediated signaling pathway”, “regulation of programmed cell death”, “oxidoreductase activity”, and “protein kinase activity” were the main enriched terms in the blue module (**Supplementary Figure S3A** and **Supplementary Table S5**). KEGG enrichment analysis indicated that “Phenylpropanoid biosynthesis”, “MAPK signaling pathway”, “Biosynthesis of secondary metabolites”, “Plant-pathogen interaction”, “Brassinosteroid biosynthesis”, and “Flavonoid biosynthesis” were the main significantly enriched metabolic pathways, indicating that these genes in blue module involved in the LSI reaction in *C. oleifera* by regulating “Phenylpropanoid biosynthesis”, “MAPK signaling pathway”, “Biosynthesis of secondary metabolites”, “Plant-pathogen interaction”, “Brassinosteroid biosynthesis”, and “Flavonoid biosynthesis” (**Supplementary Figure S3B** and **Supplementary Table S6**). In pink, statistics on GO terms showed that “tubulin binding”, “cytoskeletal protein binding”, “protein kinase regulator activity”, “cytoskeletal part”, and “microtubule cytoskeleton” were the main significantly enriched terms (**Supplementary Figure S3C** and **Supplementary Table S7**). KEGG enrichment analysis revealed that most DEGs were enriched in “Phenylpropanoid biosynthesis”, “Flavonoid biosynthesis”, “Biosynthesis of secondary metabolites”, “Ubiquitin-mediated proteolysis”, which show that genes in Pink module contribute to reject self-pollen of *C. oleifera* by regulating Flavonoid biosynthesis and Ubiquitin-mediated proteolysis (**Supplementary Figure S3D** and **Supplementary Table S8**).

### 3.7 Hub genes and TFs involved in the LSI of *C. oleifera via* WGCNA

**Figure 5:**
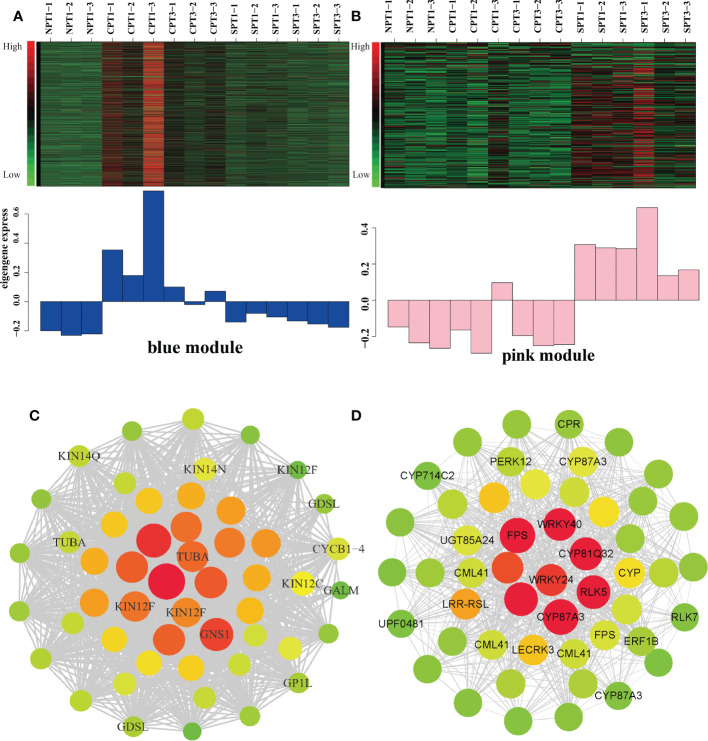
Analysis of the blue and pink modules. **(A)** Expression pattern in blue modules under different pollination treatments. **(B)** Expression pattern in pink modules under different pollination treatments. **(C)** Correlation network of the blue module. Each node represents a gene, the edge between genes represents the coexpression correlation, and the top 50 genes ordered by degree with high connectivity were visualized by Cytoscape. The color and size of each circle represent the number of edges. **(D)** Correlation network of the pink module. Each node represents a gene, the edge between genes represents the coexpression correlation, and the top 50 genes ordered by degree with high connectivity were visualized by Cytoscape. The color and size of each circle represent the number of edges.

To fur identify the specific gene s and the regulatory relationship within the modules related to LSI in the blue and pink modules, the top 10% of edges, which are ordered by the edge weight coefficient, were selected to create the gene coexpression network by CYTOSCAPE software (**Figures 5A–D** **;** **Supplementary Tables S9**, **S10**). After deleting the unknown genes, the top 50 genes ordered by degree with high connectivity are shown in **Figure 5** **(** **Supplementary Tables S11**, **S12**). From our results, we indeed discovered some important genes that might be related to LSI in Camellia oil trees. The blue modules were significantly positively correlated with FSR under self-pollination. Five genes encoding *CML41*, *LECRK3*, *CRP*, *P450* (*CYP87A3*), *WRKY*, and *ERF1B* were identified as candidate hub genes based on the degree values (**Figures 5A, C**). It has been reported that the apical Ca^2+^ gradient has an important influence on the growth of pollen tubes, and the calcium-binding protein (*CML*) protein plays a key role in the Ca^2+^ signaling system (Zhao et al., 2020). In heteromorphic SI, cytochrome *P450* (*CYP*) is one of five important S-locus genes that can determine the length of the style (Zhao et al., 2022). *ERF1B* is an ethylene-responsive transcription factor that plays a key role in the ethylene signaling pathway, and it has been reported that *ETH* is related to PCD of pollen tube. *LECRK3* encodes a G-type lectin S-receptor-like serine/threonine-protein kinase and is considered to be involved in the SI signal transduction mechanism in tea plant (Ma et al., 2018). In the pink modules, *KIN12F* and *GDSL* were identified as candidate hub genes (**Figures 5B, D**), which were significantly negatively correlated with the fruit setting rates of Camellia oil tree. *KIN12F* encodes a kinesin-like protein that can regulate on several important stress-responsive genes, such as ethylene-responsive transcription factors and RNA polymerases. (Islam et al., 2020). The *GDSL* esterase/lipase protein is vital for anther and pollen development (Yuan et al., 2020). The result indicated that these hub genes play a positively role in the LSI reaction according to the gene correlation networks.

Moreover, we also found that several genes were related to the LSI of *C. oleifera* in the pink and blue modules, although the connectivity degree was not in the top 50. Four DEGs encoding F-box proteins were identified and had relatively high expression levels in CPT1 and CPT3 (**Figure 3C**). The F-box is the pollen S-determinant in GSI, and SLF from different S-haplotypes in pollen can protect pollen tube growth from its own *S-RNase*, resulting in the breakdown of incompatibility (Zhao et al., 2022). Four DEGs encoding *RALF* were identified in these two modules, and their expression in cross-pollination was significantly higher than that in self-pollination. *RALF* and *AGP* are involved in normal pollen tube (PT) elongation in the style and PT-ovular guidance (Seth et al., 2019). The expression levels of *ATL6*, *DURF2*, *ICR4*, *SIZ1* and *ERD4*, which are three types of E3 ubiquitin ligases related to ubiquitin-mediated proteolysis, were significantly higher in cross-pollination than in self-pollination. Moreover, 1 calcium-dependent protein kinase gene, *CPK5*, and 3 probable calcium-binding protein *CML41* genes were identified to be involved in plant-pathogen interactions. Overall, these genes were identified as candidate genes related to the inhibition of pollen tube growth in pistils, revealing the molecular mechanism of the LSI of *C. oleifera*.

**Figure 6:**
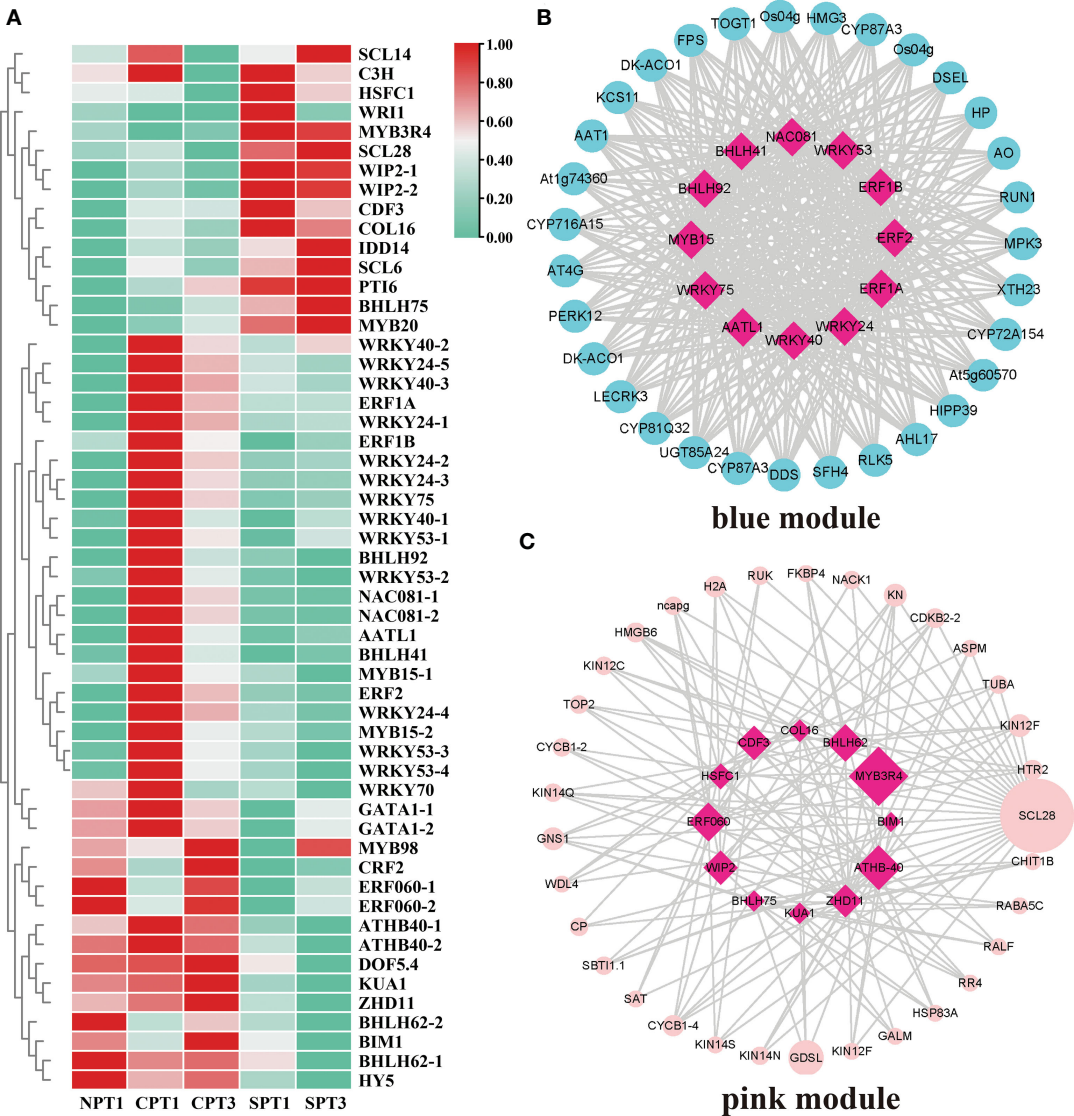
Analysis of TFs in the blue and pink modules. **(A)** Heatmap showing the expression profiles of the TFs in the self- and cross-pollination treatments. **(B)** Genetic regulatory networks (GRNs) between hub genes in blue modules. **(C)** Genetic regulatory networks (GRNs) between hub genes in the pink module.

Various TFs and their regulated gene expression networks play vital roles in plant growth, biosynthesis of secondary metabolites and development (Wu et al., 2020a). In this study, 597 TFs were identified among all of the DEGs (**Supplementary Table S13**). The *MYB*, *ERF*, *bHLH*, *WRKY* and *C2H2* families were the top 5 TF families, and the *MYB* family was one of the largest families, containing 83 members. However, members of the *SRS*, *WOX*, *RAV*, *LFY*, FA*R1*, *YABBY* and *GeBP* families had fewer than 4 genes. To systematically study the TFs and their regulatory relationship with hub genes related to the LSI of *C. oleifera*, a total of 54 TFs were obtained in the pink and blue modules, and a heatmap of their expression patterns was presented in **Figure 6A**. By calculating the correlation coefficient between TFs in modules and hub genes, and then screening the related pairs as edge information based on the correlation coefficient (blue module:|correlation coefficient| ≥ 0.9, p < 0.05; pink module: |correlation coefficient| ≥ 0.7, p < 0.05; **Supplementary Tables S14**, **S15**), we constructed an inferred regulatory network to explore the regulatory relationship between the TFs, hub genes and genes related to LSI in pink or blue modules. In the blue module, 12 hub TFs mainly in the *WRKY*, *NAC*, *MYB*, *ERF*, *bHLH*, and *FAR1* families showed regulatory relationships with hub genes (**Figure 6B**). It has been reported that the WRKY TF family played an important role in pollen germination, pollen viability and pollen tube growth in several plants (Zou et al., 2010; Guan et al., 2014), such as cotton (Wang, 2019) and *Arabidopsis* (Guan et al., 2014). In addition, a regulatory relationship between hub TFs and hub genes was also observed in the pink module (**Figure 6C**), including the TF with the highest connective degree, *MYB3R4*, which was involved in the SI process (He et al., 2020). More importantly, other TFs (*ATHB-40*, *BHLH62* and *ERF060*) associated with the stimulus response were also detected in the regulatory relationship network. The results suggested that TFs in two modules might be pivotal regulators in the LSI response of *C. oleifera*, but this finding needs further verification.

### 3.8 Alternative splicing events analysis during the LSI reaction in *C. oleifera*

**Figure 7:**
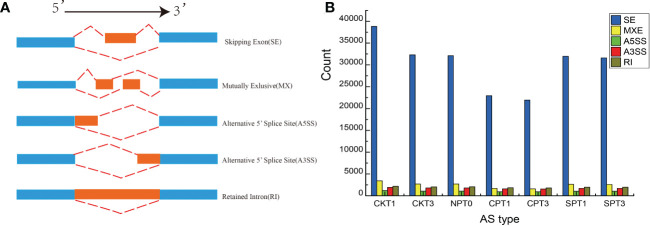
AS analysis of *C.oleifera* ovaries. **(A)** Visualization of seven AS types. **(B)** Numbers of different AS events detected in transcriptomes from ovaries.

Alternative splicing (AS) is an important gene regulation mechanism in eukaryotic plants. In this study, five AS events were detected, including: skipping exon (SE), retained intron (RI), mutually exlusion exon (MXE), alternative 5′ splice site (A5SS), and alternative 3′ splice site (A3SS) in the ovary of C. oleifera **(** **Figure 7A** **)**. As shown in the **Figure 7B**, 72,299 AS events were identified, and among which SE was the most abundant, accounting for 82.43% of AS events. RI (3.22%), together with A3SS (3.09%), A5SS (2.02%) or MXE (9.24%), these four types of AS events only accounted for (17.58%) **(** **Figure 7B** **)**. To further investigate whether the AS types with different functional annotations exhibited bias in self- and cross- pollination, the GO and KEGG pathway analyses were performed. The SE isoforms were mainly enriched in the GO terms “salicylic acid metabolic process”, “gene expression”, “response to chemical” and “signal transduction”. KEGG revealed that “Plant-pathogen interaction”, “Ubiquitin mediated proteolysis”, “Endocytosis” and “Plant hormone signal transduction” were the main pathways for the AE-type transcripts. For A3SS-type transcripts, “developmental process involved in reproduction” was the most enriched GO term, and significant pathways of “SNARE interactions in vesicular transport”, “Ubiquitin mediated proteolysis”, and “RNA transport” were identified in A3SS type transcripts. The Metabolic pathways was significantly enriched in three AS types including A3SS-, MEX- and RI-type transcripts.

Among the DEGs identified that might be involved in the LSI reaction of *C. oleifera* in this study, approximately 25 DEGs exhibited SE-type alternative splicing events, including *F-Box*, *CYP87A3*, E3 ubiquitin-protein ligase *RGLG2-like*, G-type lectin S-receptor-like serine *CES101*, *WRKY40*, and *BHLH75*. Two types of AS both had one DEG each, the A3SS-type gene *CSLD5* and the A5SS-type gene *AK2*. Two DEGs, *BHLH75* and callose synthase 2-like isoform X1, exhibited MXE-type alternative splicing events. Three DEGs, including *CDC20-1* and 2 *WRKY* transcription factors, exhibited RI-type alternative splicing events. Overall, the results suggested that the DEGs identified in this study, which potential involved in the LSI reaction, undergo variable splicing events, it enhanced the plasticity of transcriptome and also might play a role in regulating the level of different transcripts.

### 3.9 Quantitative real-time PCR validation of RNA-Seq data

**Figure 8:**
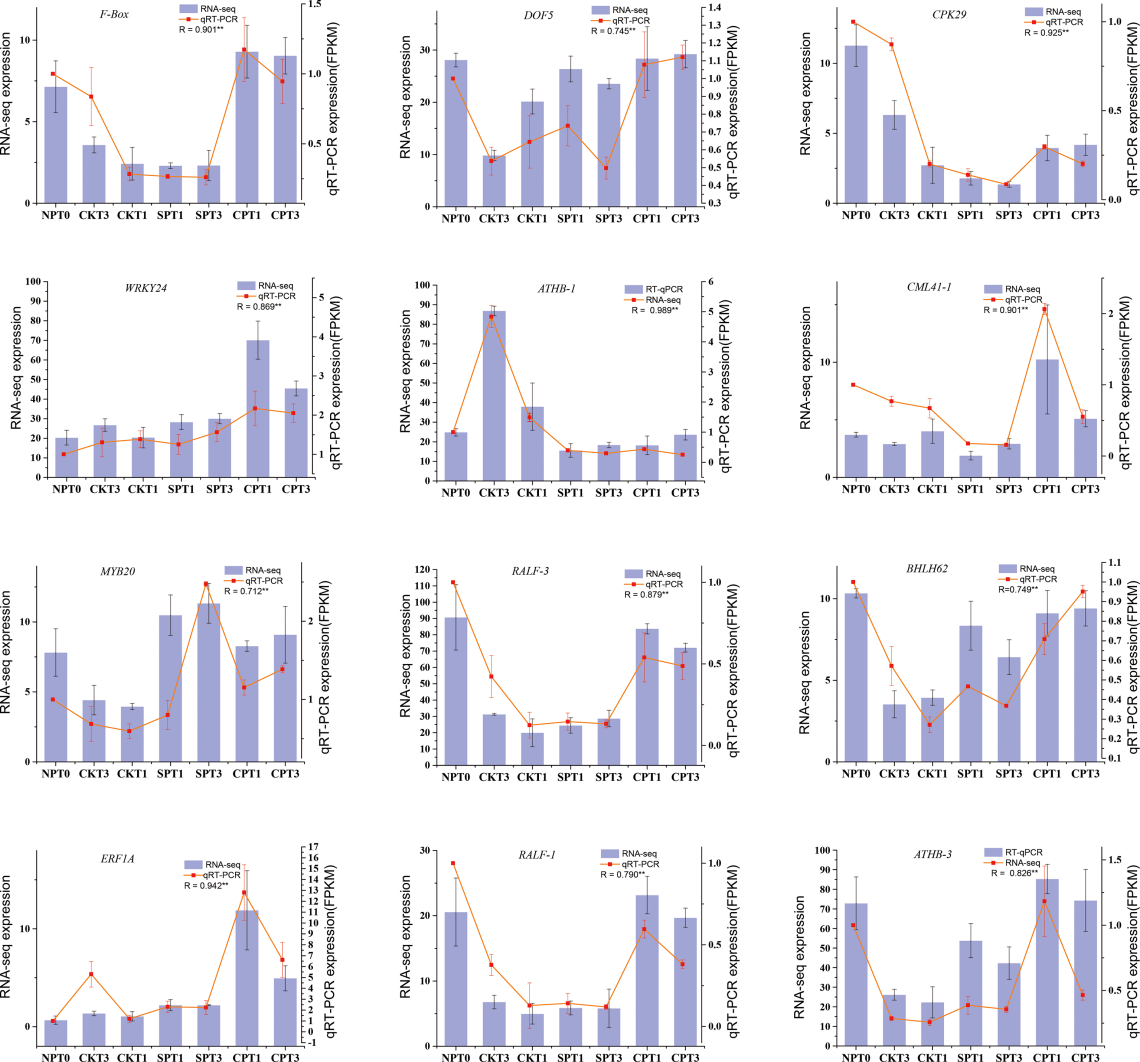
qRT-PCR validation of 12 DEGs identified as related to self-incompatibility. *p < 0.05; **p < 0.01.

To validate the reliability of our RNA-seq data, a total of 12 DEGs mentioned in this study were selected and subjected to qRT–PCR. Among the 12 DEGs, *F-Box* (ID: oil_tea_GLEAN_10305490) was related to ubiquitin-mediated proteolysis. *RALF-1* (ID: MSTRG.114451), *RALF-3* (ID: MSTRG.55710) and *CAML41* (ID: oil_tea_GLEAN_10201557) were related to the growth of pollen tube. The other 8 genes were transcription factors involved in LSI reaction of *C. oleifera*, including *DOF5* (ID: oil_tea_GLEAN_10115754), *CPK29* (ID: oil_tea_GLEAN_10394599), *WRKY24* (ID: oil_tea_GLEAN_10391421), *ATHB-1* (ID: oil_tea_GLEAN_10231301), *MYB20* (ID: oil_tea_GLEAN_10287798), *BHLH62* (ID: oil_tea_GLEAN_10050733), *ERF1A* (ID: oil_tea_GLEAN_10347617), *ATHB-3* (ID: oil_tea_GLEAN_10174212). We compared the expression data of the 12 DEGs obtained by qRT-PCR and RNA-seq, and the expression patterns of these 12 selected DEGs obtained by qRT-PCR were corresponded well with RPKM values obtained by RNA-seq (**Figure 8**). The experiment demonstrated that the RNA-seq data used in this study were highly reliable.

## 4 Discussion

The oil of Camellia oil tree seed has been used as a high-quality edible oil and a traditional medicine in China (Cheng et al., 2015). However, the yield of *C. oleifera* seeds is affected by the climate and by its LSI biological characteristics (He et al., 2020). The molecular mechanism of SI has been widely studied in plants (Zhao et al., 2015; Ma et al., 2018; Shin et al., 2022), but little is known about the SI of *C. oleifera*. In this study, we found that self-pollination at earlier bud developmental stages increased the fruit setting rate in *C. oleifera* to a certain extent. The present study reported the fruit setting rates of different treatments with self-and cross-pollination at early bud developmental stages, and the molecular regulatory mechanism of Camellia oil tree was preliminarily revealed by transcriptomic analysis. The results led to a focus on some hub genes and vital pathways, including plant MAPK signaling pathways, ubiquitin-mediated proteolysis, plant-pathogen interaction, vital signal molecules and TFs, which are discussed below.

### 4.1 Pollination at different bud developmental stages affects the fruit setting rate

It has been reported that the incongruity in SI or interspecific crossing could be overcome by bud pollination in breeding programs (Goldman, 2015). In *brassica*, pollination using mature pollen was performed on immature flowers (3–4 DBF) to bypass the influence of SI (Hiscock and Dickinson, 1993). In S-RNase-based GSI plants, it was reported that the degree of SI was positively correlated with the content of S protein in the style, and the content of S protein in the style was lower in the bud stage and higher in mature flowers (Anderson et al., 1986; McClure et al., 1999; Roldán et al., 2010). Therefore, many self-incompatible plants were self-pollinated in the immature bud stage to create homozygous offspring. In this study, the fruit setting rate of SPT3 (3–4 DBF) was higher than that of SPT1 (1 DBF), but it did not reach a significant level, suggesting that the fruit setting rate might be higher in the case of self-pollination in the earlier developmental stage of flower buds.

Effective pollination with a high fruit setting rate is determined by multiple factors, including pollen tube kinetics, stigma receptivity, and ovule development (Sanzol and Herrero, 2001). In the cross-pollination comparison, the fruit setting rate of CPT3 was lower than that of CPT1. Studies have shown that the developmental stage of the style could influence the number of growing pollen tubes, and mature stigmas have more energy and nutrients to maintain more pollen growth (Goldman, 2015), which is also an important reason for the lower fruit setting rate in cross-pollination with immature stigmas.

### 4.2 F-box and ubiquitin-mediated proteolysis are involved in the LSI of *C. oleifera*

Protein hydrolysis mediated by ubiquitination plays an important role in the GSI reaction, and this mechanism was first proposed in the study of SI in petunia (Zhang et al., 2016; Chen et al., 2018; Ren et al., 2020; Zhao et al., 2021). In self-incompatible *Petunia* and *Antirrhinum* species, *S-RNase*, which is the S-determinant of pistils, could be recognized by pollen S-determinant SLFs and ubiquitinated by forming functional *SCF* complexes in a nonself-recognition manner and then degraded by the 26S proteasome pathway, restricting the cytotoxicity of *S-RNase* and allowing pollen tubes to grow normally (Qiao et al., 2004; Hua and Kao, 2006; Hua et al., 2008; Kubo et al., 2010; Liu et al., 2014). *F-box*, *Cullin-1* (*CUL1*), S-phase kinase-associated protein 1 (*Skp-1*), and RING box protein-1 (*Rbx1*) are the functional components of the *SCF* complex (Hua and Kao, 2006). In this study, we found that the two key modules (pink and blue), which had a high correlation with fruit setting rates, were both significantly enriched in the ubiquitin-mediated proteolysis pathway, including three genes, *RZPF34*, *CDC20-1*, and *UBC20*. In addition, we also found 4 DEGs encoding F-boxes and three types of E3 ubiquitin ligases related to ubiquitin-mediated proteolysis in self- and cross-pollination. Research has shown that ubiquitin ligase can reduce the production of reactive oxygen species (ROS) and cell death, positively regulating plant salt tolerance (Potocký et al., 2007). Furthermore, we identified nine RNase T2 family genes in the self-pollination transcriptome data of *C. oleifera* Huashuo in our previous study. The discovery of these DEGs encoding F-box and ubiquitin-mediated proteolysis proteins implied that the LSI in *Camellia* oil tree might be under gametophytic control, and the enhanced understanding of the mechanism needs further verification.

### 4.3 TFs and hub genes related to LSI selected by WGCNA

The LSI reaction in *C. oleifera* is not controlled by a single gene. It is a rather a complex genetic network containing multiple genes. It has been reported that TFs play an important role in plant biological processes by regulating relative gene expression, including plant growth, synthesis of secondary metabolites (Song et al., 2022), stress/stimulus responses (Luo et al., 2022), flower senescence (Ji et al., 2022), SI reaction processes (Hou et al., 2022), pollen tube growth (Zou et al., 2010) and other important biological processes. In tomato, progressive mutation of TFs led to a transition from outcrossing to self-pollination (Benoit, 2021). In this study, 54 TFs were identified by WGCNA that were involved in the LSI process in the two key modules, and they were significantly differentially expressed. Among these TFs, *WRKY*, *MYB* and *bHLH* were the major TFs with high connectivity degrees to other hub genes. *WRKY* TFs have been demonstrated to regulate the plant response to biotic stress, such as salicylic acid (SA)-mediated defense and disease responses (Dellagi et al., 2000; Asai et al., 2002; Lai et al., 2008). In *Arabidopsis*, *WRKY34* has been demonstrated to be a pollen-specific TF and is also a substrate of *MPK/MPK6* (Guan et al., 2014). Moreover, MAPK signaling pathways were significantly enriched in both the SPT1 vs. CPT1 and SPT3 vs. SPT3 comparisons. *MYB* is a large TF family in plants, and it has been reported that *MYB* TFs are associated with the response to external or internal stimuli and plant development (Ito, 2005). *MYB* TFs are also involved in regulating several important activities associated with female reproduction in *Arabidopsis* (Punwani et al., 2008). In addition, the *bHLH* TFs *JAZs* and *MYB* could form a complex that may play an important role in seed production and stamen development in *Arabidopsis* (Qi et al., 2015). The results suggested that the three major transcription factors (*WRKY*, *MYB* and *bHLH*) might play an important role in the transcriptional activation of genes involved in the LSI reaction in *C. oleifera*.

The pollination and fertilization of plants can be divided into four stages, namely, pollen germination, tube growth, tube guidance and double fertilization (Dresselhaus and Franklin-Tong, 2013). We observed that the pollen tubes of *C. oleifera* stopped growing at the bottom of the style, where cell–cell communication events should take place at the late stage of pollen tube growth, just before double fertilization. The rapid alkalinization factor (*RALF*) is a ligand−receptor pair with *FERONIA* (*FER*). The *RALF*-*FER* pathway can mediate cell elongation (Zhu et al., 2021), and it also plays a key role in communication between the pollen tube and the synergid cell during pollen tube reception (Li et al., 2016; Liang and Zhou, 2018; Ge et al., 2019). In this study, we identified two *RALF* genes in the pink module that were expressed at significantly higher levels in cross-pollination than in self-pollination. Moreover, two hub genes were identified in the blue module: *UTG* and G-type *lecRLK*. A study in *Arabidopsis* suggested that the mutation of *EVAN* and *TURAN* led to overgrowth of pollen tubes inside the female gametophyte without rupture of pollen tubes. *UTG* superfamily proteins are encoded by *TURNA*, which is very important for pollen tube growth and integrity by affecting the stability of pollen-specific *FERONIA RLKs* (Escobar-Restrepo et al., 2007; Lindner et al., 2015). Our results suggested that the low expression of *RALF*, *UTG* and G-type *lecRLK* in self-pollination treatments was one of the main reasons why pollen tubes stopped growing and failed to fertilize.

Cytochrome *P450* has wide-range catalytic activity and is involved in the synthesis and metabolism of sterols, terpenoids, fatty acids, alkaloids, isoflavones, flavonoids and plant hormones (Sun et al., 2019). In our study, three cytochrome *P450* 87A3-like genes (*CYPs*) were identified as candidate hub genes selected by WGCNA, and the expression of *CYPs* in cross-pollination was significantly higher than that in self-pollination. Furthermore, the flavonoid biosynthesis pathway was significantly enriched in both the blue module and pink module. Since it has been reported that flavonoids play an important role in flower development, especially in the growth of pollen tubes (Lan et al., 2017; Paupière et al., 2017), studies in maize and petunia suggested that pollen deficient in flavonoids could not produce functional pollen tubes, while the defect can be overcome by applying specific flavonols to the stigma during pollination (Hosokawa et al., 2013; Han et al., 2016). In addition, DEGs in the brassinosteroid biosynthesis pathway were significantly enriched in both the SPT1 vs. CPT1 and SPT3 vs. CPT3 comparisons. In *Primula*, brassinosteroids have been demonstrated to regulate pistil development and SI reactions (Manghwar et al., 2022). *CYP734A50* was reported to be a key S-locus gene, and its products can degrade brassinosteroids (Zhao et al., 2019). In this work, how *CYP*, flavonoids and brassinosteroids work together during fertilization in *Camellia* oil trees remained unknown, but we suggested that these genes might be involved in the LSI reaction in *C. oleifera*. In addition, the qRT–PCR results demonstrated the accuracy of our transcriptomic data analysis.

**Figure 9:**
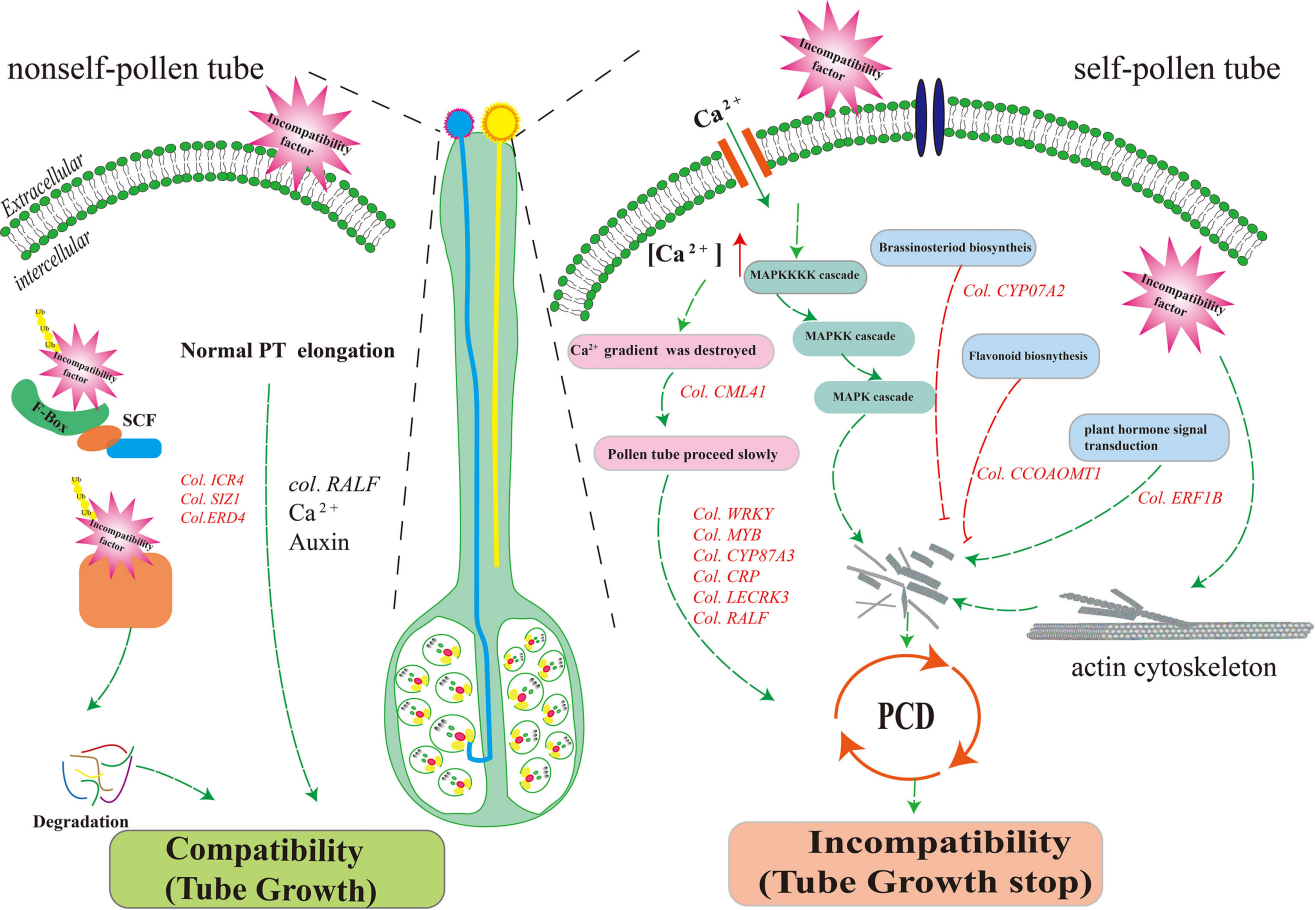
Proposed model of the LSI reaction in *C. oleifera* ovaries.

In this study, we identified a set of candidate genes and major enrichment metabolic pathways related to self-incompatibility, we developed a hypothetical model based on the results. When the self- and nonself-pollens fall on the stigma of Camellia oil tree, both can germinate and grow normally. When the pollen stretches to the base of the style and the upper part of the ovary, the self-incompatible factor enters the pollen tube to exercise the function of cytotoxicity, degrades the pollen tube RNA by the way of ubiquitinated protein hydrolysis, and starts a series of downstream molecular signal transmissions, and at the same time, plants themselves can also resist damage from self-pollination by regulating flavonoid biosynthesis, plant MAPK signaling pathways, ubiquitin-mediated proteolysis, and plant-pathogen interaction pathways. While finally the pollen tube stopped growth and programmed cell death was occurred (**Figure 9**).

## 5 Conclusion

First, transcription analysis of early-development self- and cross-pollinated ovaries was performed in *C. oleifera*. The MAPK signaling pathway, ubiquitin-mediated proteolysis, flavonoid biosynthesis, brassinosteroid biosynthesis, and plant–pathogen interactions were significantly enriched during the pollination process. Through WGCNA, we confirmed hub genes and TFs involved in the LSI reaction in *C. oleifera.* Thus, our study provided a useful transcriptomic resource for revealing the molecular mechanism of LSI in *C. oleifera* and also provided a solid theoretical basis for comprehensively increasing the output of *C. oleifera.*

## Data availability statement

The original contributions presented in the study are included in the article/**Supplementary Material**. Further inquiries can be directed to the corresponding authors.

## Author contributions

Methodology, CL, SW, JZ and XT. Data analysis, CL, YL. Data curation, YL. Resources, YL and YX. Validation, CL and YL. Writing-review and editing, CL, ML and JZ. Writing-original manuscript, CL. Supervision, JZ, SW and XT All authors have read and agreed to the published version of the manuscript.
