# Supplementary figures and images for: Gene coexpression analysis reveals key pathways and hub genes related to late-acting self-incompatibility in Camellia oleifera
Source: Front Plant Sci. 2023 Jan 24;13:1065872. doi: 10.3389/fpls.2022.1065872 (PMC9902722; doi:10.3389/fpls.2022.1065872)

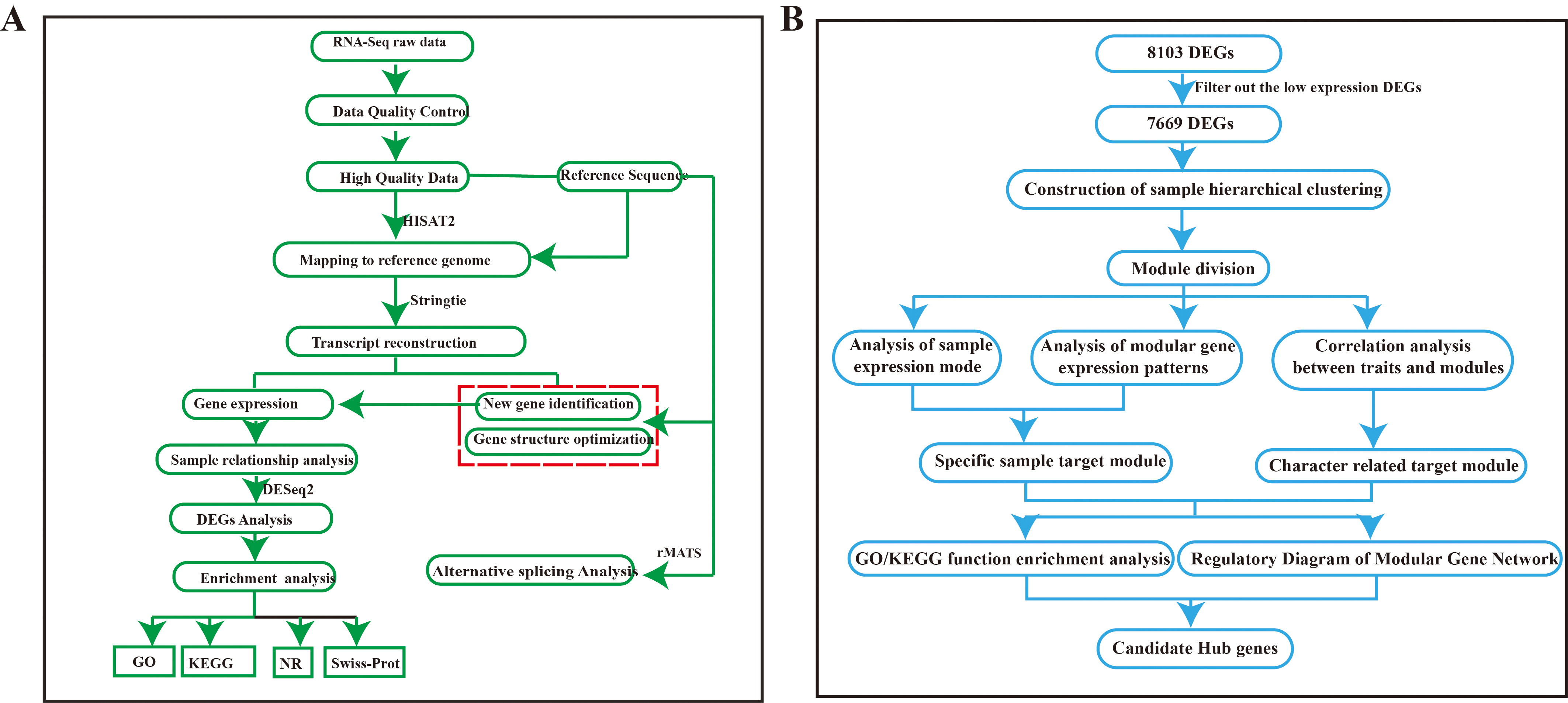

Supplement: Supplementary file 1 [file Image_1.jpeg]

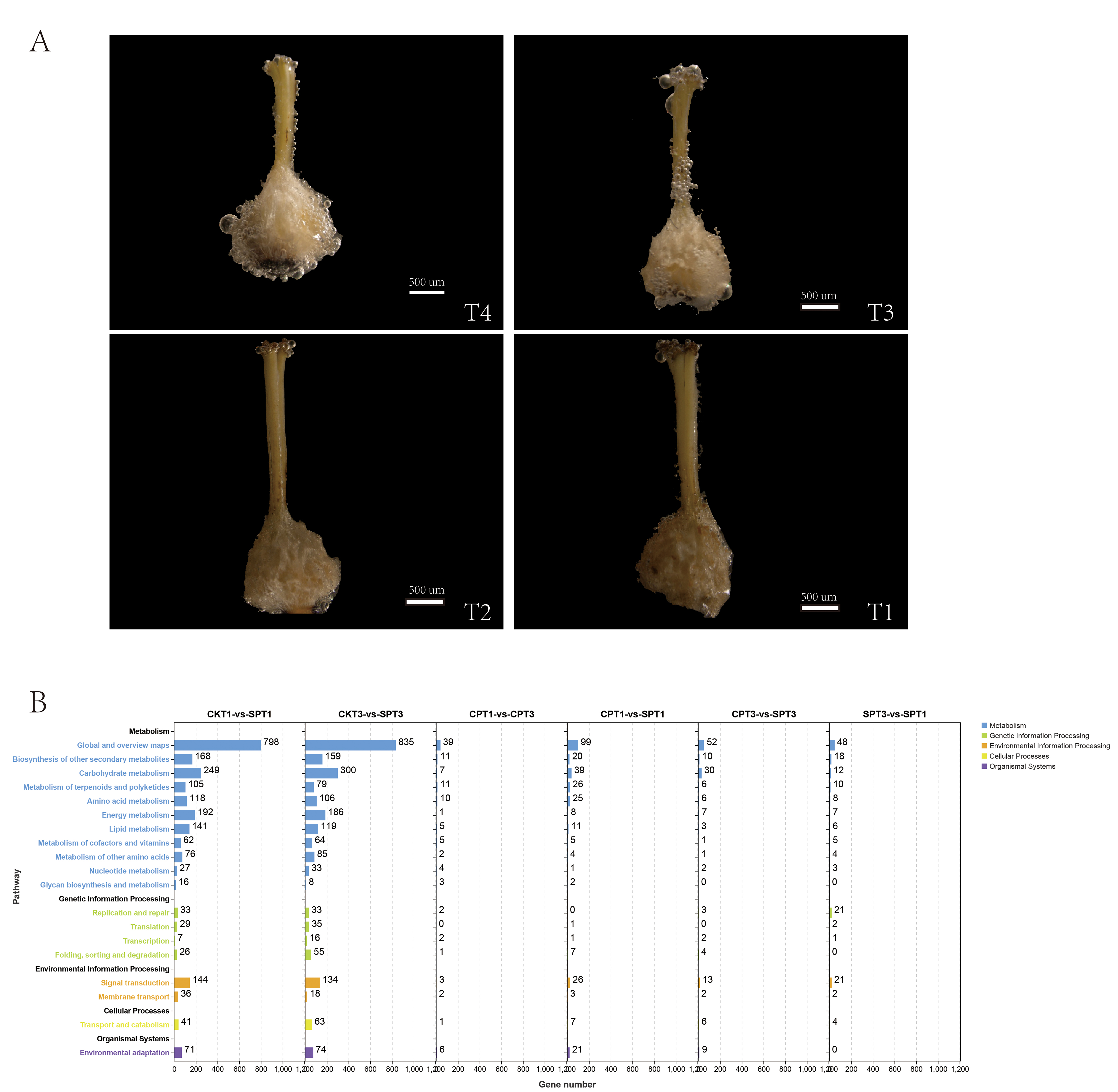

Supplement: Supplementary file 2 [file Image_2.jpeg]

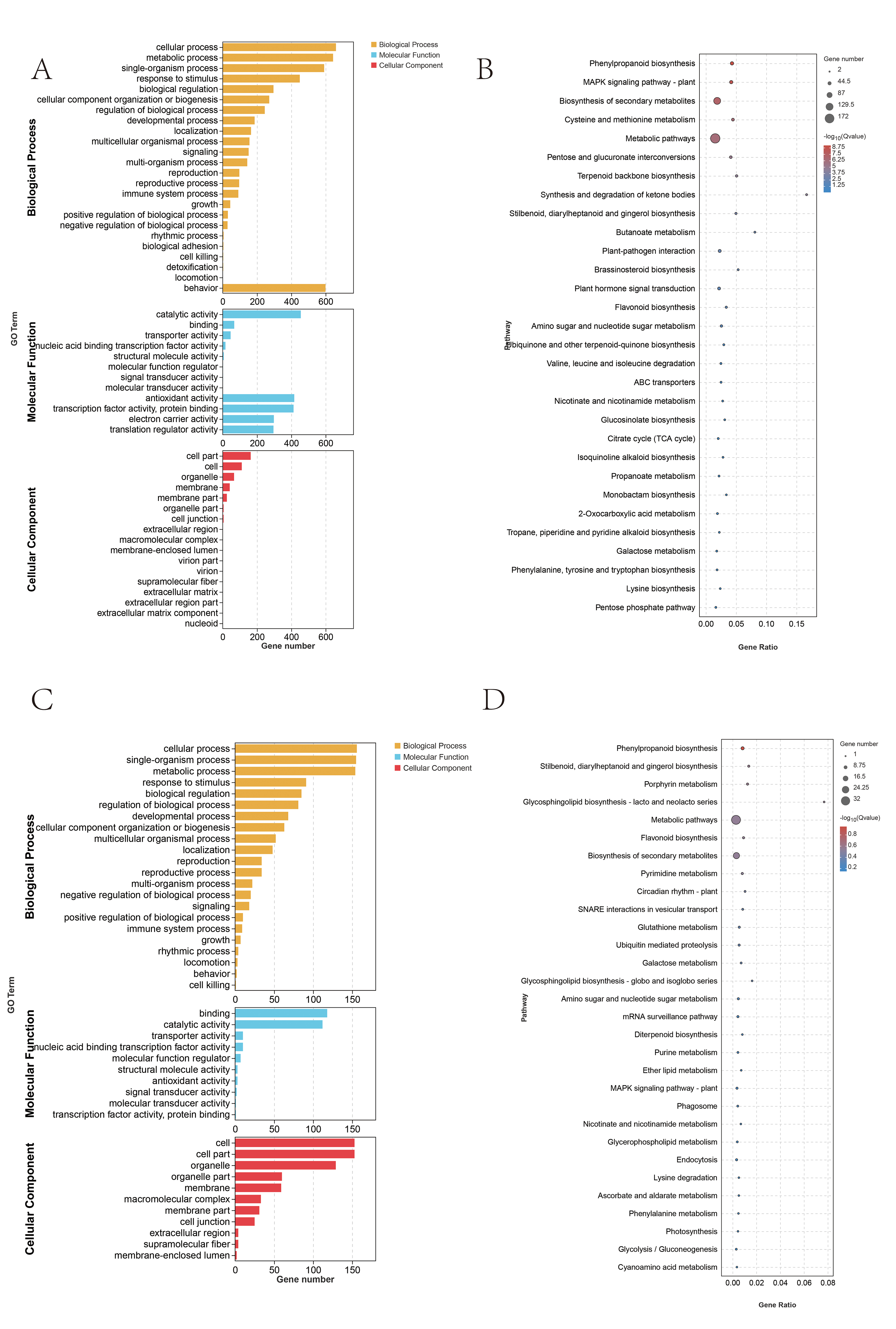

Supplement: Supplementary file 3 [file Image_3.jpeg]
